# CINENet: deep learning-based 3D cardiac CINE MRI reconstruction with multi-coil complex-valued 4D spatio-temporal convolutions

**DOI:** 10.1038/s41598-020-70551-8

**Published:** 2020-08-13

**Authors:** Thomas Küstner, Niccolo Fuin, Kerstin Hammernik, Aurelien Bustin, Haikun Qi, Reza Hajhosseiny, Pier Giorgio Masci, Radhouene Neji, Daniel Rueckert, René M. Botnar, Claudia Prieto

**Affiliations:** 1 School of Biomedical Engineering and Imaging Sciences, King’s College London, St. Thomas’ Hospital, Lambeth Wing, London, UK; 2 Department of Computing, Imperial College London, London, UK; 3 MR Research Collaborations, Siemens Healthcare Limited, Frimley, UK; 4 Escuela de Ingeniería, Pontificia Universidad Católica de Chile, Santiago, Chile

**Keywords:** Computational models, Data acquisition, Image processing, Machine learning, Biomedical engineering, Computational science, Magnetic resonance imaging, Three-dimensional imaging, Cardiology

## Abstract

Cardiac CINE magnetic resonance imaging is the gold-standard for the assessment of cardiac function. Imaging accelerations have shown to enable 3D CINE with left ventricular (LV) coverage in a single breath-hold. However, 3D imaging remains limited to anisotropic resolution and long reconstruction times. Recently deep learning has shown promising results for computationally efficient reconstructions of highly accelerated 2D CINE imaging. In this work, we propose a novel 4D (3D + time) deep learning-based reconstruction network, termed 4D CINENet, for prospectively undersampled 3D Cartesian CINE imaging. CINENet is based on (3 + 1)D complex-valued spatio-temporal convolutions and multi-coil data processing. We trained and evaluated the proposed CINENet on in-house acquired 3D CINE data of 20 healthy subjects and 15 patients with suspected cardiovascular disease. The proposed CINENet network outperforms iterative reconstructions in visual image quality and contrast (+ 67% improvement). We found good agreement in LV function (bias ± 95% confidence) in terms of end-systolic volume (0 ± 3.3 ml), end-diastolic volume (− 0.4 ± 2.0 ml) and ejection fraction (0.1 ± 3.2%) compared to clinical gold-standard 2D CINE, enabling single breath-hold isotropic 3D CINE in less than 10 s scan and ~ 5 s reconstruction time.

## Introduction

Cardiac CINE magnetic resonance imaging (MRI) is the gold standard for the assessment of cardiac morphology and function. Conventionally, multi-slice 2D CINE imaging is performed under multiple breath-holds to achieve left ventricular (LV) coverage. For fast LV coverage only a few (~ 12) short-axis 2D slices with anisotropic resolution in the slice direction are acquired throughout multiple breath-holds of < 15 s duration each. Imperfect (e.g. drifts) or varying breath-hold positions and the anisotropic image resolution can cause slice misalignments which may lead to staircasing artifacts and erroneous assessment of the ventricular volume. The LV function assessment is assessed by epicardial and endocardial segmentation of the images in short-axis orientation. Indeed, the anisotropic resolution of the short-axis 2D CINE does not allow for reformats to arbitrary orientations. Further images in other long axis orientations are required for a comprehensive assessment of cardiac morphology and function which in turn requires multiple acquisitions to be performed in several geometric views and thereby increasing overall planning and scan time.

To overcome these limitations, 2D^1,2^ and 3D^3–5^ free-breathing cardiac CINE imaging with retrospective motion correction have been proposed to minimize slice misalignment and improve patient comfort. Data are acquired under free-breathing and respiratory and cardiac motion is resolved retrospectively which comes however at the expense of a prolonged scan time in the order of several minutes. Moreover, these approaches usually require long reconstruction times associated with the high-dimensional (spatial, respiratory temporal and cardiac temporal) data processing or with the nature of their sampling trajectory during data acquisition.

Shorter cardiac CINE acquisitions can be achieved if the respiratory motion does not need to be resolved or corrected. Single breath-hold 2D real-time acquisitions^6,7^ or 2D simultaneous multi-slice (SMS) for cardiac imaging^8,9^ have been studied for this purpose, but provide only limited LV coverage and are still hampered by anisotropic image resolution in the slice direction. To increase the LV coverage, reconstruction of pseudo 3D cardiac CINE datasets from multiple multi-slice anisotropic 2D volumes by using motion-corrected super-resolution frameworks have been proposed^10,11^. This requires however several low-resolution scans (in different orientations) in the order of several minutes and depend on slice-to-volume registration accuracy.

LV coverage with higher spatial resolution can be obtained with single breath-hold 3D cardiac CINE^12–17^. The fast single breath-hold acquisitions require acceleration by parallel imaging (PI)^18^ and/or compressed sensing (CS)^19^ which leverage the multi-channel receiver coil information and/or spatio-temporal redundancies of the image. However, in case of PI, maximal achievable acceleration is limited by the amount of MR receiver coils. In case of CS, the maximum acceleration is limited by the selected undersampling during acquisition, the prior information and the selected reconstruction technique. These limitations lead to a trade-off in the acquisition between spatial and temporal resolution for a given LV coverage.

Generally, PI methods allow for accelerated acquisitions by a factor of 2–3× without sacrificing image quality. PI is therefore widely used in clinical applications within these acceleration limits. CS allows for a stronger sub-Nyquist sampling if (1) the images are compressible, i.e. can be sparsely represented in some transform domain, if (2) are incoherently sampled, i.e. for cardiac CINE meaning incoherent undersampling within and between cardiac phases and if (3) a non-linear reconstruction is employed. Reconstructions can be accomplished with iterative algorithms that use a fixed sparsity-promoting transformation^20^ or that adaptively derive the optimal sparse representation from the data themselves, known as dictionary learning^21^.

High image quality was obtained with previously proposed 3D cardiac CINE methods by trading off spatial resolution in the slice direction thereby preventing high-resolution reformats in arbitrary views. Anisotropic slice resolution in the range of 2.5–10 mm within a single breath-hold of 10–27 s^12–16^ was obtained for accelerations of up to 21× (depending on sampling trajectory). We have recently proposed a 3D Cartesian cardiac CINE MRI acquisition which achieves isotropic resolution in a single breath-hold of ~ 20 s with full LV coverage^17^. The proposed 3D CINE can be acquired in non-oblique orientation (e.g. sagittal) reducing planning time while providing high-resolution reformats in any arbitrary view, such as short-axis or long axis. While this extended breath-hold of ~ 20 s is feasible in healthy subjects, it is in general too long for most patients. Moreover, the relatively long reconstruction time of this approach (~ 5 min) can limit its clinical adoption. For widespread usage in clinical routine, breath-hold durations should be in the range of a conventional multi breath-hold 2D CINE of < 15 s and reconstruction times should be in the order of few seconds. Further reduction of the breath-hold duration is therefore desirable but can only be achieved with an increase in undersampling if no compromise is made on spatial or temporal resolution.

For the desired higher undersampling factors, fixed sparsity assumptions in CS are often too restrictive and incapable of fully modelling the spatio-temporal cardiac dynamics. Careful fine-tuning between regularization and data consistency is required and especially in highly undersampled cases residual aliasing may remain in the image or over-regularization can occur leading to staircasing or blurring artifacts. Moreover, previously proposed reconstruction techniques^22^ are computationally demanding and require significant long reconstruction times, rendering it complicated to be integrated into clinical workflow. Recently, deep-learning based reconstruction methods have gained attention to solve these non-linear and ill-posed optimizations efficiently^23–25^. Proposed methods range from derivations of classical optimizations (e.g. ADMM-Net)^26^, over cascaded convolutional networks^27–29^, UNet-based convolutional networks^30,31^ and recurrent neural networks^32^ to generative adversarial network-based denoising (e.g. DAGAN)^33,34^, manifold learning^35^, variational neural networks^36–38^ and generalized PI reconstructions^39–41^. Network inputs differ from single-coil 2D magnitude image^27–29^ and/or k-space^26,27,35^ to multi-coil 2D magnitude/phase image^31,34,37,41^, 2D k-space^29,30,39,40,42,43^ or low-resolution 3D k-space^44^ and were studied for static imaging^24,26,27,29–31,33–38^, i.e. no temporal dynamics, or for 2D dynamic imaging^28,32,41^, i.e. 2D + time such as 2D cardiac CINE, that handle complex-valued data as separate real/magnitude and imaginary/phase channels^28,30,36,38,41^ or networks^31^.

In this work, we propose a novel multi-coil complex-valued 4D (3D + time) deep-learning based MR reconstruction for highly prospectively undersampled 3D Cartesian cardiac CINE data. The proposed CINENet enables acquisition of single breath-hold 3D CINE with 1.9mm^3^ isotropic LV coverage in less than 10 s scan time and ~ 5 s reconstruction time. The network is trained on in-house acquired 3D Cartesian cardiac CINE data of an electrocardiogram (ECG) triggered balanced steady-state free-precession sequence using a variable-density Cartesian trajectory with spiral-like order (VD-CASPR). The proposed 4D CINENet exploits spatio-temporal redundancies by cascaded and complex-valued (3 + 1)D spatial and temporal convolutions with complex-valued processing and handling of multi-coil data via data-derived coil sensitivity maps. The architecture resembles an unrolled proximal gradient algorithm with sparsity-learning and data consistency steps^17^. CINENet is evaluated on prospectively undersampled 3D Cartesian cardiac CINE data of 20 healthy subjects and 15 patients undergoing a clinically referred cardiac MR protocol. CINENet is compared against a CS reconstruction and to the clinical gold-standard 2D CINE sequence qualitatively and quantitatively in terms of LV function assessment and contrast ratio between myocardium and blood pool.

## Results

A fourfold cross-validation with training on 15 healthy subjects and validation on 5 held-out subjects was conducted. Further testing included 3D cardiac CINE from 15 patients with suspected cardiovascular disease. We trained CINENet by retrospectively undersampling the isotropic 3D CINE reference data (iterative SENSE reconstruction of 2.5× prospective undersampled acquisition) of healthy subjects with randomly selected acceleration factors in the range of 3× to 8× and temporal resolutions per cardiac phase in the range of 16–78 ms, resulting in 6,500 4D volumes. Undersampling masks follow a VD-CASPR sampling with incoherent sampling between and within cardiac phases^17^. We performed a supervised training with voxel-wise mean-squared error loss between reconstructed image of CINENet and iterative SENSE reconstructed reference. The proposed CINENet consisting of sparsity-learning and data fidelity blocks resulted in ~ 2.1 million trainable parameters with spatial convolutional kernels of size 5 and temporal kernels of size 3 and a fixed data fidelity weighting of 0.01. The convolutional kernels achieved a spatial and temporal receptive field coverage of 33% and 100% of the input image, respectively. In terms of computational efficiency, we report an average training duration of ~ 35 min/epoch, i.e. ~ 24 h in total for ~ 40 epochs on 2 GPUs (Nvidia Titan RTX) and we observed an average reconstruction time of ~ 5 s for CINENet and ~ 2 min for a GPU-accelerated CS.

**Figure 1:**
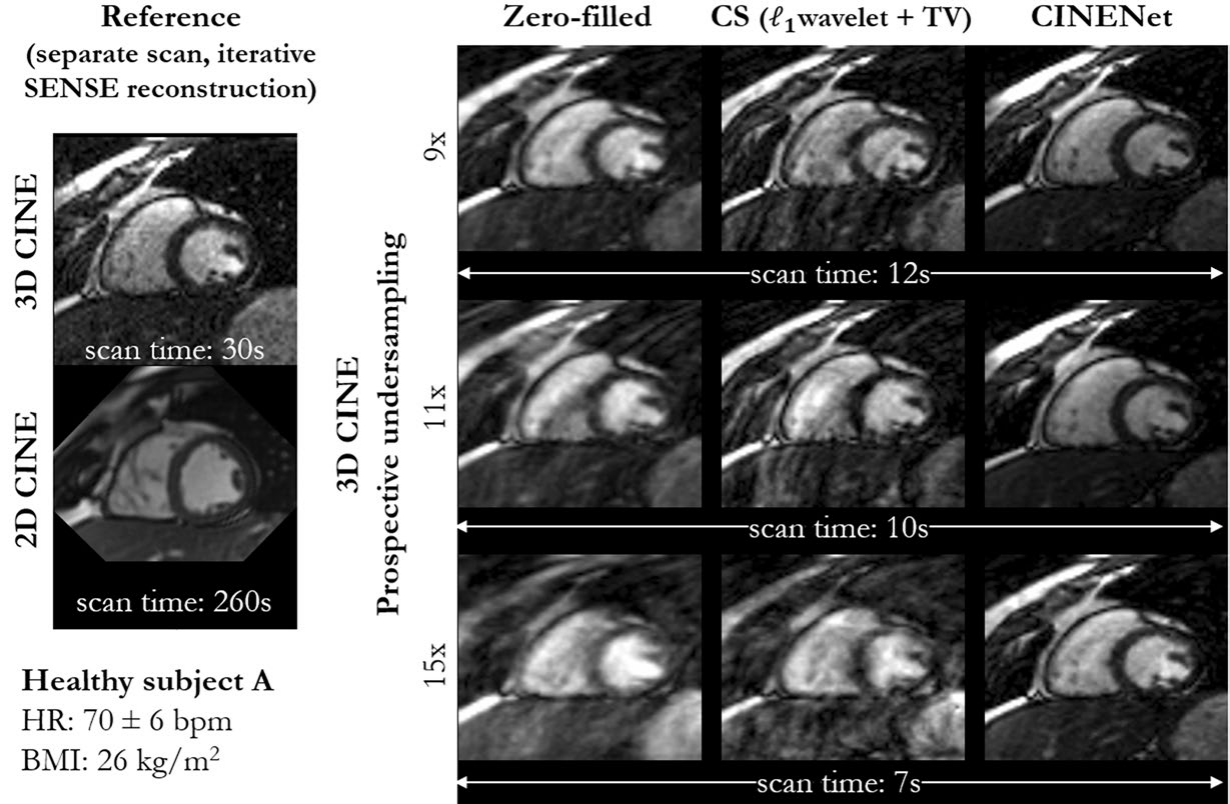
End-diastolic, mid-apical images in short axis of a healthy subject with strong fat-related aliasing. Images are acquired with prospectively undersampled single breath-hold 3D Cartesian CINE with isotropic (1.9 mm^3^) left ventricle coverage for an acceleration of 9× (scan time 12 s), 11× (scan time 10 s) and 15× (scan time 7 s) in comparison to ground-truth reference (separate acquisition) of single breath-hold 3D CINE (2.5×, scan time 30 s) and conventional multi breath-hold 2D CINE (2×, scan time 260 s, 15 s acquisition and 20 s pause per breath-hold). The reference 3D CINE is reconstructed with iterative SENSE. Undersampled 3D CINE images are reconstructed with coil-weighted zero-filling (network input), Compressed Sensing (CS) with L1-regularized spatial wavelets and temporal total variation (TV) and with the proposed CINENet. Supplementary Video S1 depicts the cardiac motion-resolved images.

**Figure 2:**
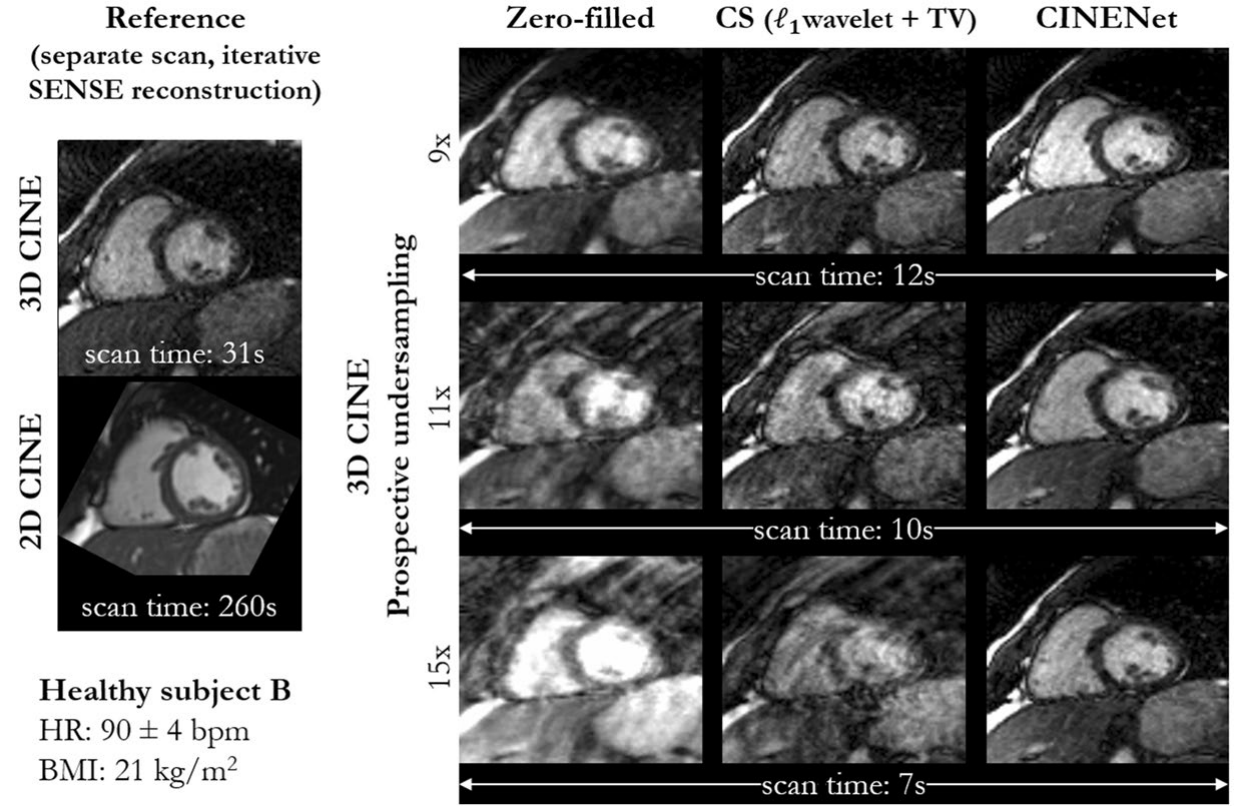
End-diastolic, mid-apical images in short axis of a healthy subject with elevated heart rate. Images are acquired with prospectively undersampled single breath-hold 3D Cartesian CINE with isotropic (1.9 mm^3^) left ventricle coverage for an acceleration of 9× (scan time 12 s), 11× (scan time 10 s) and 15× (scan time 7 s) in comparison to ground-truth reference (separate acquisition) of single breath-hold 3D CINE (2.5×, scan time 30 s) and conventional multi breath-hold 2D CINE (2×, scan time 260 s, 15 s acquisition and 20 s pause per breath-hold). The reference 3D CINE is reconstructed with iterative SENSE. Undersampled 3D CINE images are reconstructed with coil-weighted zero-filling (network input), Compressed Sensing (CS) with L1-regularized spatial wavelets and temporal total variation (TV) and with the proposed CINENet.

Figures 1, 2, Supplementary Fig. 1 and Supplementary Video S1 depict mid-apical images in short axis of healthy subjects with varying heart rate and body mass index which are affected by different levels of fat-related aliasing. Images were acquired with single breath-hold isotropic 3D Cartesian cardiac CINE and reconstructed with different techniques. A coil sensitivity weighted zero-filled image, CS with ${\mathcal{l}}_{1}$-regularized spatial wavelets and temporal total variation (TV)^13^ and the proposed CINENet reconstructions are shown for separate prospectively undersampled acquisitions with 1.9 mm^3^ isotropic LV coverage of 9×, 11× and 15× acceleration corresponding to a scan time of 12 s, 10 s and 7 s. The zero-filled reconstruction corresponds to the network input. A separate reference acquisition (2.5×) with an iterative SENSE reconstruction was performed in an extended breath-hold of ~ 30 s to appreciate achievable image quality. Conventional multi breath-hold 2D CINE (2×) with a scan time of 260 s (15 s acquisition and 20 s pause per breath-hold) and resolution of 1.9 × 1.9 × 8 mm^3^ is depicted as clinical gold-standard in a similar short axis slice.

Visually improved reconstruction results for 3D CINE are obtained with the proposed CINENet compared to CS, suggesting that spatio-temporal redundancies are better exploited with the proposed approach. Moreover, subject-adaptive learning seems to outperform fixed regularized CS even when subject-specific CS parameters are used, as exemplary illustrated in Fig. 1 and Supplementary Fig. 2. Especially for highly accelerated datasets (> 9×), the proposed CINENet still achieves good image quality whereas aliasing and blurring artifacts are still present in the CS reconstruction. High visual agreement to conventional 2D CINE is achieved with CINENet. The proposed 3D CINE achieved high temporal resolution per cardiac phase of ~ 45 ms.

**Figure 3:**
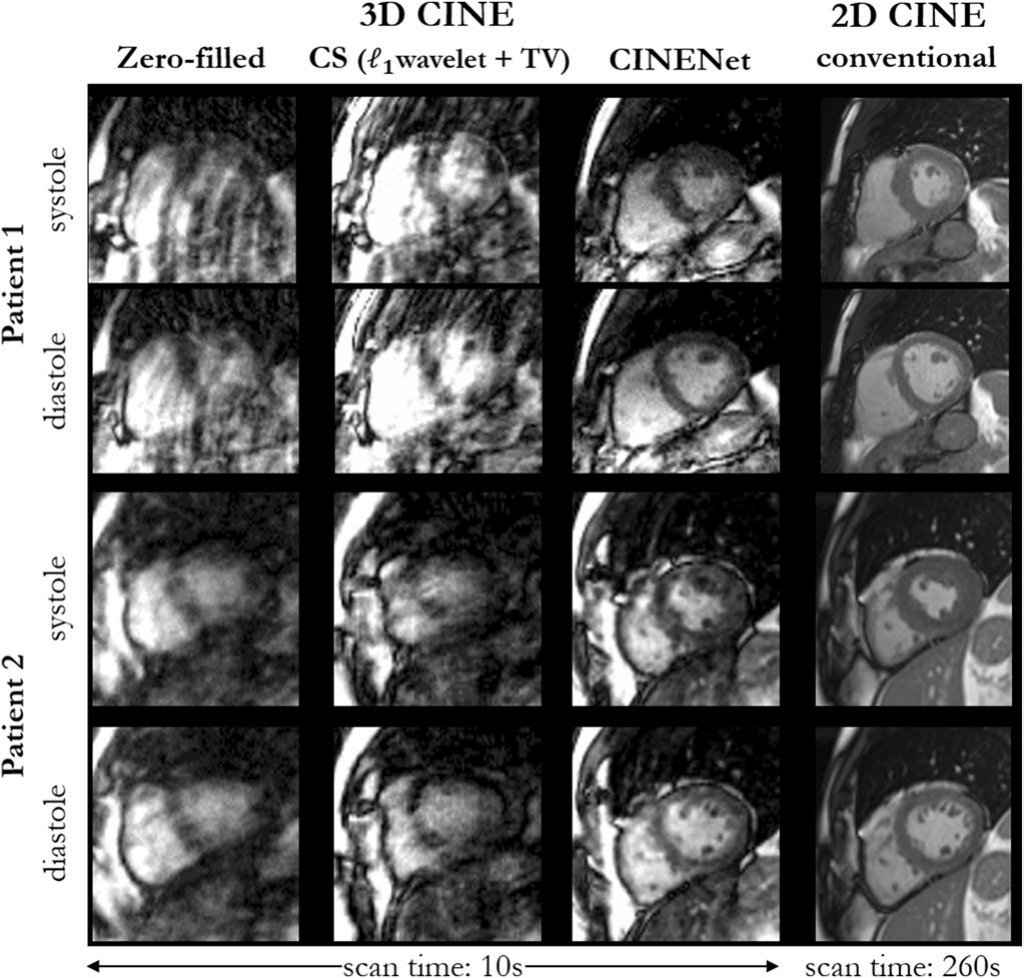
End-systolic and end-diastolic mid-apical images in short axis of two patients with suspected cardiovascular disease (Patient 1: arrhythmogenic right ventricular cardiomyopathy, Patient 2: arrhythmogenic right and left ventricular cardiomyopathy). Images are acquired with prospectively undersampled single breath-hold 3D Cartesian CINE with slightly anisotropic (1.9 × 1.9 × 2.5 mm^3^) left ventricle coverage for an acceleration of 12× (scan time 10 s) in comparison to conventional multi breath-hold 2D CINE (2×, scan time 260 s, 15 s acquisition and 20 s pause per breath-hold). Undersampled 3D CINE images are reconstructed with coil-weighted zero-filling (network input), Compressed Sensing (CS) with L1-regularized spatial wavelets and temporal total variation (TV) and with the proposed CINENet.

In Fig. 3 and Supplementary Fig. 3, we show qualitative reconstruction results for four patients of systolic and diastolic mid-apical slices in short axis from the proposed 4D CINENet. A slightly anisotropic 3D CINE acquisition of 1.9 × 1.9 × 2.5 mm resolution and 12× acceleration (scan time 10 s) was acquired and reconstructed with coil-weighted zero-filling (network input), CS with ${\mathcal{l}}_{1}$-regularized spatial wavelets and temporal TV and CINENet. In the patient population, we encountered myocarditis, arrhythmogenic right and left ventricular cardiomyopathy, restrictive cardiomyopathy, dilated cardiomyopathy, hypertensive cardiomyopathy, non-ischaemic cardiomyopathy, embolic myocardial infarction and eosinophilic granulomatosis with polyangiitis (EGPA) with cardiac involvement.

As illustrated in Fig. 3, while CS reconstruction suffers from strong aliasing (patient 1) and TV-induced staircasing artifacts (patient 2), the proposed CINENet mitigates artifacts and recovers high-frequency details, such as papillary muscles. Reconstructions of CS and CINENet are in higher visual accordance in patient 3 and 4 (Supplementary Fig. 3), but sharper delineation between myocardium and blood pool is achieved with CINENet. In comparison to conventional multi breath-hold 2D CINE, the 3D CINE reconstructed with CINENet shows good agreement but with the advantage of a 22-fold shorter acquisition time that can be achieved within a single breath-hold.

**Figure 4:**
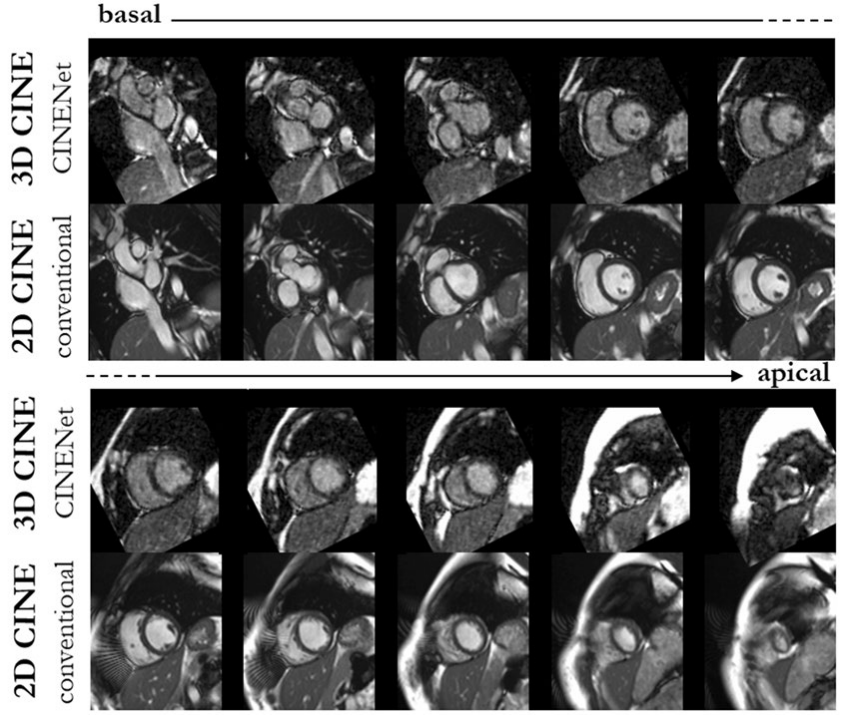
End-diastolic images in short axis ranging from base to apex of one healthy subject acquired with 15× accelerated single breath-hold 3D Cartesian CINE (scan time 7 s) and reconstructed with the proposed 4D CINENet in comparison to conventional multi-slice and multi breath-hold 2D CINE (scan time 260 s). For 3D CINE, similar anatomical slice locations to 2D CINE have been selected, i.e. not all 3D CINE slices are shown. Supplementary Video S2 depicts the cardiac motion-resolved images and Supplementary Video S3 depicts the spatial coverage.

The spatial LV coverage is shown in Fig. 4 for one healthy subject in diastole, comparing basal to apical slices at similar positions of 3D CINE reconstructed with CINENet to conventional 2D CINE, i.e. not all short axis slices for 3D CINE are depicted. Isotropic LV coverage in a single breath-hold of 7 s (15×) is shown with CINENet providing high spatial and temporal resolution. All cardiac phases for the same subject are illustrated in Supplementary Video S2. LV coverage with isotropic resolution of 3D CINE in comparison to 2D CINE is shown in Supplementary Video S3.

We show temporal profiles at mid-ventrical position in Supplementary Fig. 4 for two healthy subjects and two patients of the prospectively undersampled 3D CINE (15× for healthy subjects and 12× for patients) in comparison to 2D CINE. Substantial reduction in aliasing artifacts and recovery of temporal trace was found for CINENet reconstruction. Temporal behavior of 3D CINE was in good agreement with 2D CINE. The isotropic acquisition allows reformats in arbitrary orientations. Supplementary Fig. 5 shows reformats in vertical long axis (2 chamber), horizontal long axis (4 chamber) and coronal orientation of the short axis acquisition of healthy subject A.

We show training and validation performance of the proposed 4D CINENet using (3 + 1)D complex-valued convolutions in comparison to 4D complex-valued convolutions in Supplementary Fig. 6. CINENet achieves stable convergence without overfitting and has lower training and validation loss than the network with 4D complex-valued convolutions.

**Figure 5:**
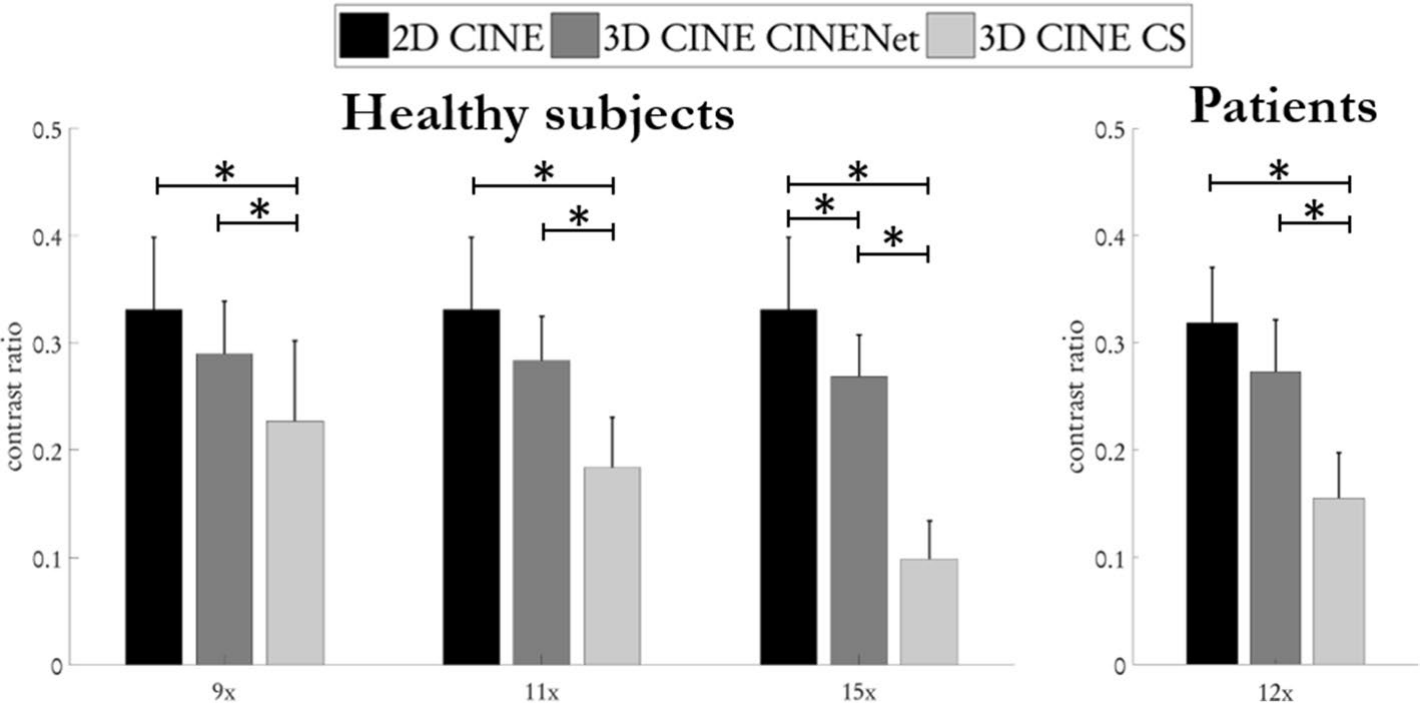
Quantitative contrast ratio analysis between myocardium and left + right ventricular blood pool averaged over apical, mid-apical and basal slices of a cohort of 20 healthy subjects and 15 patients. Comparison between conventional multi breath-hold 2D CINE, single breath-hold 3D CINE acquired with prospective undersampling of 9× (scan time 12 s), 11× (scan time 10 s), 12× (scan time 10 s) and 15× (scan time 7 s) and reconstructed with Compressed Sensing (CS) for L1-regularized spatial wavelets and temporal total variation and the proposed 4D CINENet. Average and standard deviations are depicted. Statistical differences (p < 0.05) are indicated by *. No statistical difference occurs between 2D CINE and 3D CINENet, except at acceleration factor 15×. Figure was created using Python 3.6 and Matplotlib 3.2.2^45^.

We measured the contrast ratio between myocardium and left and right ventricular blood pool in conventional 2D CINE and 3D CINE reconstructed with CINENet and CS. As illustrated in Fig. 5 in general a higher contrast ratio was obtained in conventional 2D CINE than in the prospectively subsampled 3D CINE (9×, 11×, 12×, 15×), which was not statistically significant (p = 0.10/p = 0.062/p = 0.060 for 9×/11×/12× with α = 0.05) except for 15× (p = 0.041). Contrast ratio was on average 40.0% (p = 0.001)/64.2% (p < 0.001)/284.7% (p < 0.001$$)$$ higher for healthy subjects with 9×/11×/15× in 3D CINE reconstructed with CINENet than with CS reconstructions and for patients (12×) by 85.4% (p < 0.001).

**Figure 6:**
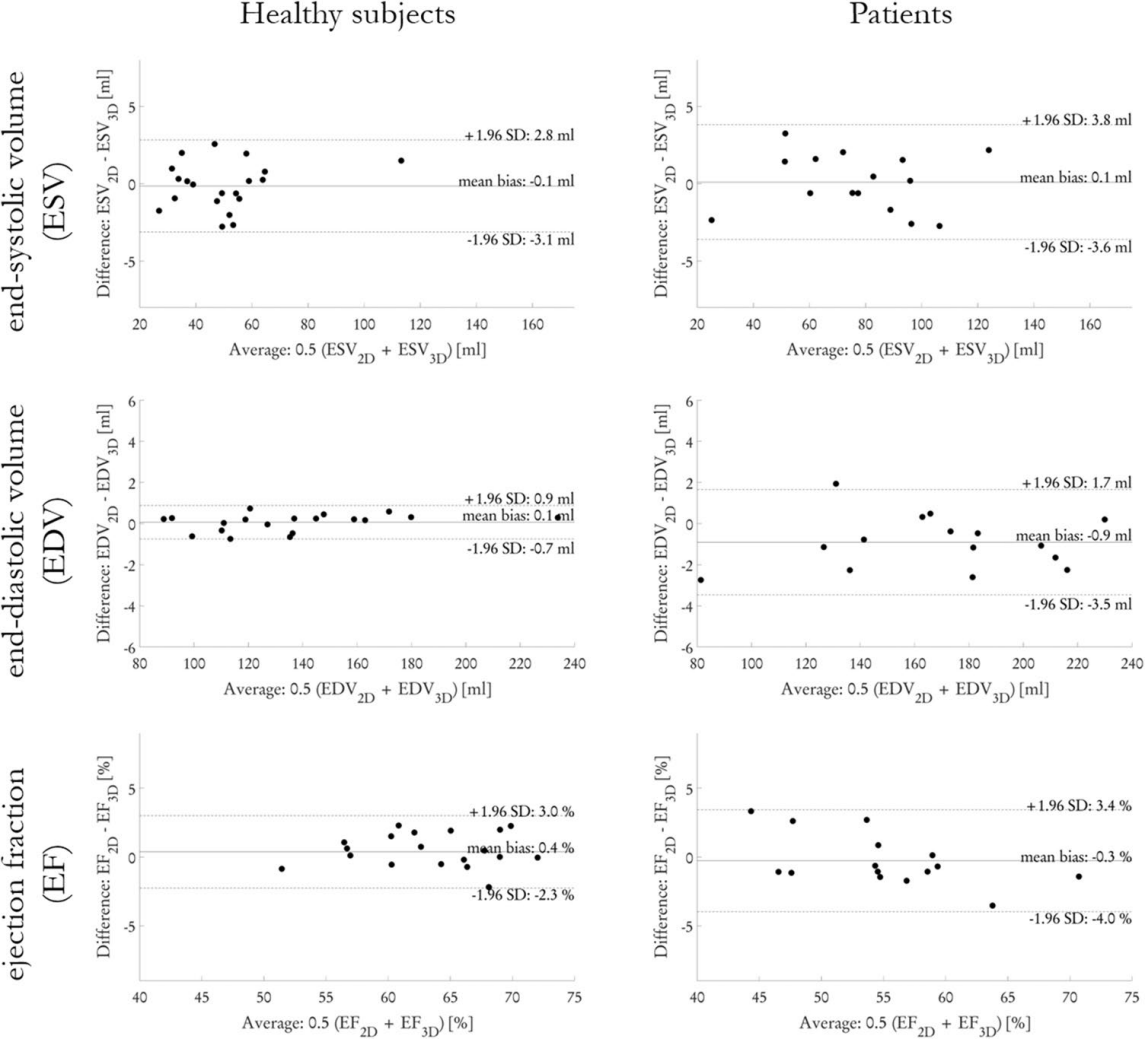
Extracted left ventricular function parameters, end-systolic volume (ESV), end-diastolic volume (EDV) and ejection fraction (EF) for isotropic (1.9 mm^3^, 15×, scan time 7 s) in 20 healthy subjects and slightly anisotropic (1.9 × 1.9 × 2.5 mm^3^, 12×, scan time 10 s) in 15 patients acquired with single breath-hold 3D CINE in comparison to conventional multi breath-hold 2D CINE. Figure was created using Python 3.6 and Matplotlib 3.2.2^45^.

Figure 6 shows the LV functional assessment in healthy subjects and patients by means of end-systolic volume (ESV), end-diastolic volume (EDV) and ejection fraction (EF). For the 3D CINE in 20 healthy subjects reconstructed with CINENet in comparison to 2D CINE, on average a bias for ESV, EDV and EF of − 0.1 ml, 0.1 ml and 0.4% was observed with all observations lying inside the confidence interval for ESV, EDV and EF of ± 3.0 ml, ± 0.8 ml and ± 2.7%, respectively. In the 15 patients, a bias for ESV, EDV and EF of 0.1 ml, − 0.9 ml and − 0.3% with all (except one for EDV) observations lying inside the confidence interval of ± 3.7 ml, ± 2.6 ml and ± 3.7%.

## Discussion

In this work, we have proposed a novel reconstruction method, named CINENet, for 3D cardiac CINE MRI based on a deep learning network which enables highly accelerated imaging sequences. We found that employing the proposed CINENet allows for 3D isotropic CINE acquisition in a single breath-hold of < 10 s scan time with ~ 5 s reconstruction time. We observed that CINENet can provide visually improved images over CS for these high acceleration factors while enabling a 22-fold faster acquisition than conventional 2D CINE and a 24-fold faster reconstruction than CS facilitating clinical translation. Our qualitative and quantitative results indicate good agreement of 3D CINE with conventional 2D CINE, which, whether confirmed in larger patient studies, will pave the way for clinically grounded applications.

Rapid assessment of cardiac function by cardiac CINE plays a vital role in patient monitoring and staging of cardiovascular or cardio-oncological diseases. Visualizing cardiac anatomy and function in 3D can help in the diagnosis of congenital heart diseases, for the detection of regional wall motion abnormalities or impaired ejection fraction. A 3D isotropic imaging resolution with Cartesian sampling allows for reformats in arbitrary orientations without loss of resolution avoiding multiple breath-hold acquisitions in double-oblique orientations. This leads to more efficient workflow and improved patient comfort.

We have reported a novel deep learning reconstruction that works with multi-coil and complex-valued 4D (3D + cardiac phases) data. The proposed architecture reflects an unrolled optimization algorithm with complex-valued convolutions and activation functions and intermittent data consistency blocks to properly handle the input data. We accomplish computationally efficient spatio-temporal information sharing by cascaded 3D spatial and 1D temporal convolutional kernels. CINENet incorporates coil sensitivity maps to provide a multi-coil reconstruction. We obtained qualitatively good images which indicates the capability of the proposed network to utilize spatio-temporal redundancies. Training for various acceleration factors and temporal resolutions improved generalizability to different inputs. We observed that the selected acceleration factors for training are sufficient to simulate the expected levels of aliasing artifact appearance. Reconstructions of prospectively undersampled acquisitions with larger acceleration factors were possible indicating enough diversity in the training database. Our results show that data-adaptive training of a sparsifying transformation for changing imaging conditions (temporal resolution, accelerations, subjects) demonstrates to be more generalizable and robust than a fixed sparsity basis and regularization parameters as in CS. In case of CS, subject- and undersampling factor-dependent regularization parameters are conceivable. Here, the selected CS regularization parameters were optimized to fit all subjects and accelerations. Furthermore, we observed that the network generalized for different field of view (FOV) placements between subjects, signal-to-noise ratio levels and mild slice resolution changes (i.e. comparing healthy subjects to patients).

Cascaded (3 + 1)D complex-valued convolutions, i.e. spatial and temporal convolutions with real and imaginary kernels and feature maps, kept trainable parameters reasonable, enabling faster and stable convergence (see Supplementary Fig. 5) while still capturing the spatio-temporal relationship^46^. A comparable implementation using full 4D complex-valued convolutional kernels, i.e. looping over combinations of 3D complex-valued convolutions, would provide a natural utilization of spatio-temporal redundancies, but would also result in ~ 80 million trainable parameters for the network even with kernel sharing amongst cardiac phases. Moreover, complex-valued convolutions enabled a natural complex data processing instead of independently handling real and imaginary parts in separate channels.

The underlying assumption of spatio-temporal sharing in 3D cardiac CINE is that it contains a rich amount of redundancy in a local neighborhood along all temporal directions (cardiac phases) which can be exploited. We assume that similar structures exist in different cardiac phases, but at different spatial locations around a certain neighborhood of a given voxel. Therefore, spatial receptive field can be limited whereas temporal coverage should span the entire cardiac cycle. Encoding and decoding branches in the 4D UNet of CINENet increase receptive fields while allowing for relatively small convolutional kernels. We achieved a receptive field coverage of 33% in spatial and 100% in temporal direction, reflecting a spatially confined but full temporal information sharing from all cardiac phases.

We used separable rectified linear (ReLU) activation functions on real and imaginary dimensions after each convolutional kernel yielding good performance and stable convergence. Further activation functions such as cardioid^47^, are conceivable but demand a careful reparameterization of the network. Batch normalization was used as it provided improved edge delineation and less blurring in the reconstructed images in comparison to instance normalization or omitting normalization layers. However, further comparisons in a larger cohort would be required to conclude the best performing combination of activation and normalization. We used SENSE-based multi-coil data consistency blocks between UNet stages to ensure data fidelity. We formulated the data consistency as proximal mapping step with a fixed regularization weighting which we can treat as a layer operation with forward and backward (gradient backpropagation) pass.

The undersampled 3D CINE data requires a joint 4D processing of the data. Previously proposed architectures for 2D dynamic imaging operate on 2D + time and are hence not applicable due to the 4D nature of the data. Furthermore, 2D CINE data cannot be leveraged as resolution, contrast, undersampled phase-encoding directions and sampling trajectories are different. Hence, visual artifact appearance will differ. If slicing of the short axis 3D images along the fully sampled frequency direction (vertical long axis) is conducted, the data would lose the 3D spatial relationship and the ability to resolve vertical long axis motion, i.e. only a few of the resulting 2D horizontal long axis slices would contain the heart and reconstruction of the dynamic through-plane (vertical long axis) motion would be impaired. Cropping of the 4D volume into smaller chunks would hence (a) loose dynamic information if cropped along temporal dimension or (b) demand consideration of band-pass filtering in data consistency^48^ implying a more complicated data fidelity block if cropped along spatial dimension. The fairest reconstruction comparison is obtained with an iterative and GPU-accelerated CS reconstruction.

Differences in contrast between 2D and 3D CINE are expected since the maximum achievable flip angle within specific absorption rate limitations of 3D CINE is lower than for 2D CINE. Moreover, saturation of the ventricle blood pool and inflow of saturated blood into the ventricle pool by the 3D slab-selective excitation affect the contrast of 3D CINE. In contrast the slice-selective 2D CINE can benefit from inflow of unsaturated blood. The contrast ratio in 3D CINE reconstructed with CINENet was on average slightly lower than for 2D CINE, but not statistically significant (except for 15×). The obtained contrast is still comparable and sufficient for reliable extraction of LV functions, but can be improved using exogenous contrast agents. We did not observe any major performance differences of the automatic LV segmentation in 2D or 3D CINE. A comparative analysis on image quality between 2D CINE and 3D CINE was conducted in our previous study^17^.

Adipose tissue, mainly observed as subcutaneous adipose tissue in the chest wall, and epicardial fat can introduce strong aliasing artifacts in highly accelerated 3D cardiac CINE. Fat suppressed acquisitions, such as water-selective excitation or fast interrupted steady-state^49–51^ can help to reduce the impact of fat, but demand longer acquisition times for similar acceleration. In this study, we were interested to enable the reconstruction to deal with de-aliasing of artifacts from adipose tissues for a non-fat suppressed acquisition. The proposed CINENet can learn the reconstruction task in the presence of fat from the paired training images of reference and undersampled input with various accelerations. In contrast, the CS reconstruction can only map the current input into a fixed sparsity domain to resolve the induced fat aliasing.

We acknowledge some limitations in this study. Imaging resolution differs in healthy subjects and patients. In patients, we used a slightly anisotropic through-plane resolution to ensure sufficient LV coverage and a conservative undersampling factor. Nevertheless, reconstruction with CINENet was not influenced by this mild resolution difference. We will address the impact of different spatial resolutions in future studies. Aliasing artifact appearance depends on the chosen sampling trajectory. We used a Cartesian acquisition with spiral profile order which provides distinct sampling patterns per cardiac frame and results in incoherent aliasing along the phase-encoding directions. Other sampling trajectories commonly seen in dynamic imaging are 3D koosh-ball or stack-of-stars^12,14,15^ which would result in streaking undersampling artifacts for which a different trained network would be probably required. Different architectural choices, such as 3D recurrent convolutional networks or variational neural networks, can be feasible which will be investigated in future studies. With the proposed framework the clinical relevance for 3D CINE over 2D CINE can be investigated in future studies.

In summary, we proposed a novel multi-coil complex-valued 4D deep learning-based reconstruction. The proposed CINENet can reconstruct highly prospectively undersampled 4D data by exploiting spatio-temporal redundancies in cascaded (3 + 1)D convolutions and enables thereby acquisition of single breath-hold 3D cardiac CINE with 1.9 mm^3^ isotropic LV coverage in less than 10 s with reconstruction times of ~ 5 s. 3D isotropic CINE enables cardiac function assessment in non-oblique orientations with reformatting in any arbitrary orientation, resulting in reduced planning and scan time and consequently improved patient comfort. Data-driven sparsity learning of CINENet outperforms fixed sparsity transformations in CS. We have found good qualitative agreement between single breath-hold 3D CINE images reconstructed with CINENet and conventional multi breath-hold 2D CINE as well as quantitative agreement in terms of LV function. The proposed 3D CINE framework can replace several consecutive 2D multi breath-hold acquisitions leading to a more efficient workflow and improved patient comfort.

## Methods

**Figure 7:**
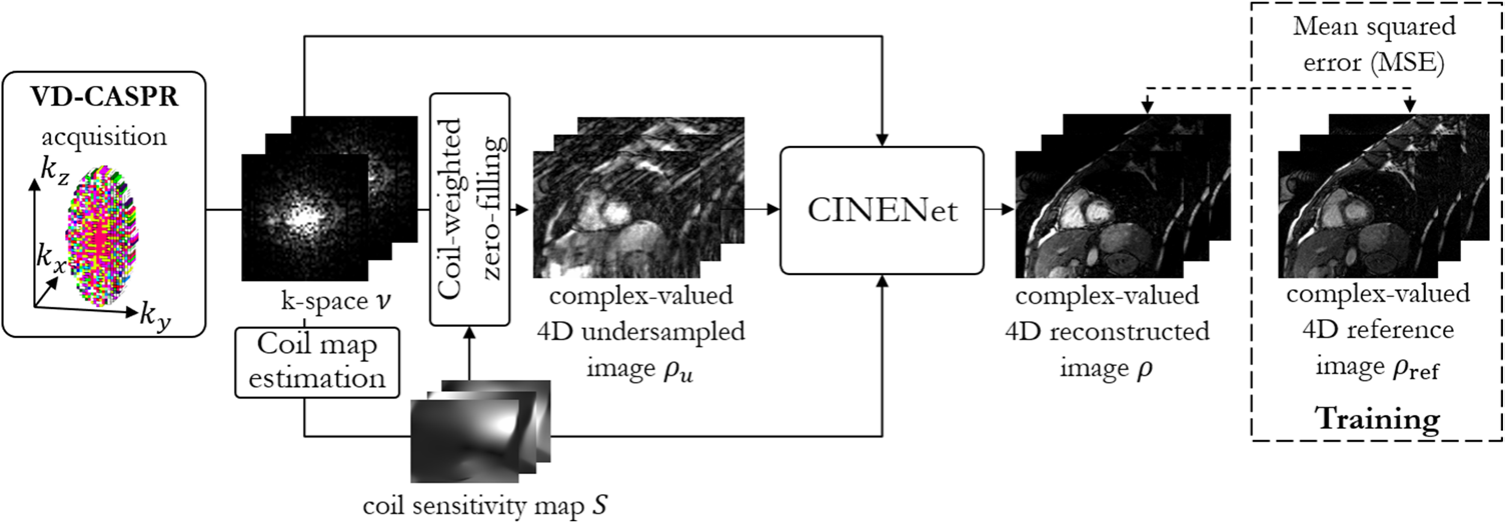
Proposed reconstruction framework: VD-CASPR subsampled k-spaces of 3D Cartesian cardiac CINE acquisition containing all *N*_*C*_ cardiac phases (different colours in the trajectory diagram) are reconstructed in the proposed 4D CINENet to the 4D cardiac motion-resolved image $\rho$. Coil sensitivity map $S$ is estimated from the low-frequency k-space data and is provided together with the k-space data $\nu$, undersampling mask and sensitivity-weighted zero-filled reconstructed image ${\rho }_{u}$ as input to the 4D CINENet. In training, the iterative SENSE reconstructed image ${\rho }_{\mathrm{ref}}$ (separate acquisition with 2.5× acceleration in a single breath-hold of ~ 30 s) is provided to guide the network training by a mean squared error (MSE) loss of the complex-valued images. Figure was created using TikZ 3.1.5.

The pipeline of data acquisition and reconstruction of this study is summarized in Fig. 7. The proposed 4D CINENet receives as input the complex-valued multi-coil k-space data $\nu$ of the 3D Cartesian CINE with VD-CASPR sampling, the coil sensitivity maps $S$ derived from k-space $\nu$ and the sensitivity weighted and coil combined undersampled 4D image ${\rho }_{u}$, to reconstruct an aliasing-free 4D image $\rho$ which is close to the reference image ${\rho }_{\mathrm{ref}}$ in a mean-squared error sense.

### Single breath-hold 3D Cartesian cardiac CINE acquisition

For 3D cardiac CINE, we utilize a VD-CASPR trajectory that undersamples the phase-encoding plane. The trajectory samples along a spiral-like arm fully sampled frequency encoding lines per repetition time. An alternating tiny-golden and golden angle increment between spiral arms and out-inward sampling provides incoherent sampling between and within cardiac phases with minimal gradient switching. One spiral arm is acquired per cardiac phase at every heart-beat using prospective ECG triggering. A fully sampled center region (15%) is acquired per cardiac phase to ensure reliable coil sensitivity map calibration^17^.

### CINENet reconstruction

**Figure 8:**
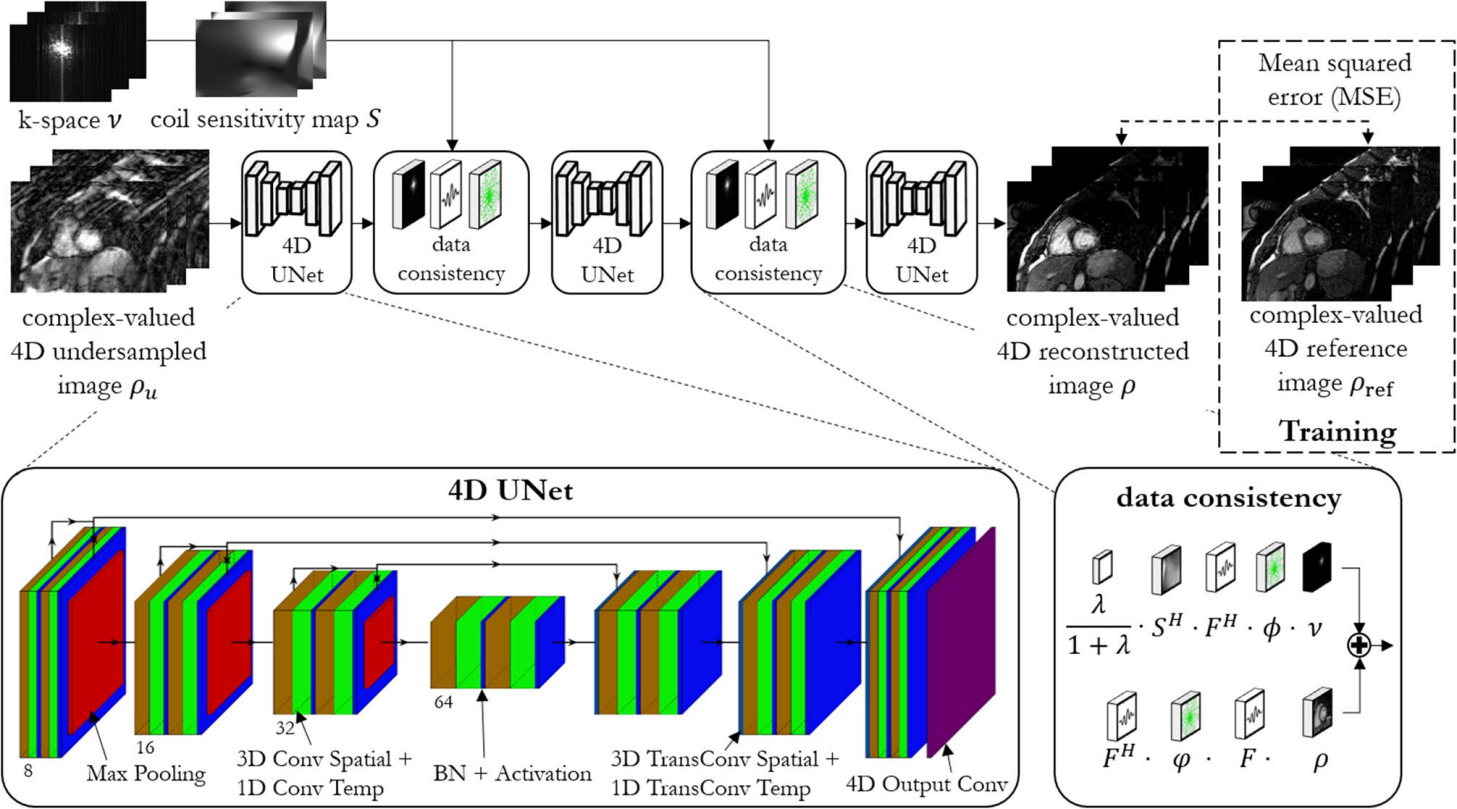
Proposed 4D CINENet resembles a proximal gradient algorithm with alternating 4D sparsity-learning UNet and data consistency blocks. The 4D UNet consist of an encoding and decoding branch with cascaded (3 + 1)D (3D + time) complex-valued convolutional layers, 3D convolution spatial (brown layer) + 1D convolution temporal (green layer), complex-valued batch normalization (BN) with rectified linear unit activation function (blue layer), max-pooling (encoder; red layer) and (3 + 1)D transposed complex-valued convolution (decoder; light blue layer). A final 4D complex-valued convolution compresses the complex-valued feature channels to a single complex-valued output channel (purple layer). Data consistency block receives as input the current reconstructed image $\rho$, the k-space $\nu$, coil sensitivity map *S*, the subsampling mask ϕ and the regularization parameter λ. $\varphi$ denotes the scaled subsampling mask, $F$ represents the Fourier transformation and ${F}^{H}$ the inverse Fourier transformation. The network is trained end-to-end with mean squared error loss between retrospective undersampled images ${\rho }_{u}$ (3× to 8× and varying temporal resolution) and iterative SENSE reconstructed reference image ${\rho }_{\mathrm{ref}}$ (2.5×). Figure was created using TikZ 3.1.5.

We propose a deep-learning based reconstruction network for 3D cardiac CINE, i.e. 3D dynamic images. The proposed 4D CINENet network is motivated by an alternating direction method of multipliers (ADMM) algorithm^17^ and is resembled as an unrolled proximal gradient algorithm with three cascaded 4D UNets and intermittent data consistency blocks (depicted in Fig. 8).

The trainable parameters of the network $\Theta$, i.e. the (3 + 1)D convolutional filters, learn the spatio-temporal sparsity during a training phase. We train the network end-to-end on pairs of images $({\rho }_{u}, {\rho }_{\mathrm{ref}})$ for which ${\rho }_{u}$ is generated by retrospectively (after acquisition) undersampling the reference image ${\rho }_{\mathrm{ref}}$ with randomly generated VD-CASPR undersampling masks $\phi$ with different acceleration factor and temporal resolution per cardiac phase. In this way we can guarantee a voxel-wise alignment of the image pairs (without the effect of remaining cardiac or respiratory motion) and can thus use a real-valued voxel-wise mean-squared error loss

1 $$L = \frac{1}{2} \left\|{ \rho - \rho_{{{\text{ref}}}} } \right\|_{2}^{2} = \frac{1}{2}\left\| {\left( {{\text{Re}}\left( \rho \right) - {\text{Re}}\left( {\rho_{{{\text{ref}}}} } \right)} \right) + \left( {{\text{Im}}\left( \rho \right) - {\text{Im}}\left( {\rho_{{{\text{ref}}}} } \right)} \right)} \right\|_{2}^{2}$$

for training where $\mathrm{Re}(\cdot )$ and $\mathrm{Im}(\cdot )$ are real and imaginary parts. Reference images are acquired with 2.5× prospective (during acquisition) undersampling and reconstructed with an iterative SENSE reconstruction^52^. Input and reference images are normalized to unit interval. Testing is then performed on prospectively undersampled data.

CINENet reconstructs an aliasing-free image $\rho \in {\mathbb{C}}^{{N}_{x}{N}_{y}{N}_{z}{N}_{t}}$ where ${N}_{x}, {N}_{y}, {N}_{z}$ describe the 3D spatial dimensions and ${N}_{t}$ the dynamic temporal cardiac phases. The input to the network is the complex-valued sensitivity weighted coil-combined zero-filled undersampled image ${\rho }_{u}\in {\mathbb{C}}^{{N}_{x}{N}_{y}{N}_{z}{N}_{t}}$ with spatial matrix size ${N}_{x}=112, {N}_{y}=160, {N}_{z}=44$ and ${N}_{t}=16$ cardiac phases, as well as the acquired k-space $\nu \in {\mathbb{C}}^{{N}_{x}{M}_{y}{M}_{z}{N}_{t}{N}_{Ch}}$ with ${N}_{Ch}=14$ receiver coils after coil compression and the VD-CASPR undersampling mask $\phi \in {\mathbb{Z}}^{M\times N}$ extracted from $\nu$, with $M\ll N, M={N}_{x}{M}_{y}{M}_{z}{N}_{t}{N}_{Ch}, N={{N}_{x}N}_{y}{N}_{z}{N}_{t}{N}_{Ch}$. The coil sensitivity maps $S\in {\mathbb{C}}^{N\times N}$ are obtained from the time-averaged fully sampled calibration center region in $\nu$ by ESPIRIT^53^ with virtual coil compression of initially 25–30 MR receiver coils (depending on field of view placement) to a common size of ${N}_{Ch}=14$.

In the reconstruction, we assume that 3D cardiac CINE images contain a rich amount of redundancy on a spatial (between and within receptive fields of a given spatial voxel) and temporal (between cardiac phases) scale. The overall unconstrained MR reconstruction problem is given by

2 $$\mathrm{arg}\underset{\rho }{\mathrm{min}}\mathcal{R}\left(\rho ;\Theta \right)+\lambda {\Vert \phi FS\rho -\nu \Vert }_{2}^{2}$$

where $F\in {\mathbb{C}}^{N\times N}$ denotes the discrete Fourier transform, ${\Vert \cdot \Vert }_{2}$ is the ${\mathcal{l}}_{2}$ norm and $\lambda >0$ is the data consistency weighting parameter. The regularizer $\mathcal{R}(\rho ;\Theta )$ is expressed by

3 $${\mathcal{R}}\left( {\rho ;{\Theta }} \right) = \left\| {\rho - f_{{{\text{CINENet}}}} \left( {\rho_{u} ;{\Theta }} \right)} \right\|_{2}^{2}$$

mapping the undersampled image ${\rho }_{u}$ to the aliasing-free output image $\rho$ via the feedforward path of the CINENet ${f}_{\mathrm{CINENet}}$. The regularizer thus minimizes the voxel-wise mean-squared error in Eq. (1). Combining Eqs. (2) and (3), we can get a closed-form solution^54^ of the unrolled proximal gradient for the reconstructed k-space

4 $$\nu^{k + 1} = \left\{ {\begin{array}{*{20}l} {Ff_{{{\text{CINENet}}}} \left( {\rho^{k} ;{\Theta }} \right),} \hfill & {\phi_{n} = 0} \hfill \\ {\frac{1}{1 + \lambda }\left( {Ff_{{{\text{CINENet}}}} \left( {\rho^{k} ;{\Theta }} \right) + \lambda FS^{H} F^{H} \phi \nu } \right),} \hfill & {\phi_{n} = 1} \hfill \\ \end{array} ,} \right.\quad \forall n \in \left[ {1,N} \right]$$

after stage/iteration $k$ for all voxels, cardiac phases and coils with $N={N}_{x}{N}_{y}{N}_{z}{N}_{t}{N}_{ch}$ which defines the data consistency layer between the sparsity-learning 4D UNet blocks.

The 4D UNet has three encoding and decoding stages with one bottleneck stage and utilizes complex-valued processing, i.e. all layers are formulated as complex-valued operations. Real and imaginary part of the data are kept in separate dimensions to perform these operations. The spatio-temporal information is exploited in the network by (3 + 1)D spatial and temporal complex-valued convolutional filters $f$ operating on the feature maps $m$ as

5 $$f*m=\mathrm{Re}\left(f\right)*\mathrm{Re}\left(m\right)-\mathrm{Im}\left(f\right)*\mathrm{Im}\left(m\right)+j\left(\mathrm{Im}\left(f\right)*\mathrm{Re}\left(m\right)+\mathrm{Re}\left(f\right)*\mathrm{Im}(m)\right)$$

Spatial convolutional layers have filter kernels of size 5 × 5 × 5 × 1 ($x\times y\times z\times t$) followed by a temporal convolution of size 1 × 1 × 1 × 3, a complex batch normalization and complex ReLU activation which acts on the real and imaginary part separately. We select a dyadic increase in channel size between stages. Residual paths within stages and between encoder/decoder stages improve convergence and avoid vanishing gradients. In the encoder branch 2 × 2 × 2 × 1 spatial max-pooling is applied between stages while transposed convolutions are performed in the decoder side. A final 4D complex-valued convolution compresses the complex-valued feature channels to a single complex-valued output channel.

Following Eq. (4), the multi-coil data consistency is given as a one-step proximal gradient which is defined in the forward pass as

6 $${f}_{\mathrm{DC}}\left({\rho }^{k}\right)=\frac{\lambda }{1+\lambda }{S}^{H}{F}^{H}\phi \nu +{F}^{H}\varphi F{\rho }^{k}$$

with the scaled undersampling mask

7 $$\varphi_{n} = \left\{ {\begin{array}{*{20}l} {1,} \hfill & {\phi_{n} = 0} \hfill \\ {\frac{1}{1 + \lambda },} \hfill & {\phi_{n} = 1} \hfill \\ \end{array} } \right.$$

We use Wirtinger calculus^55–57^ during backpropagation to update the complex-valued weights of each layer with respect to the real-valued loss function. In the backward pass due to linearity of all involved operations the gradient simplifies to

8 $$\frac{\partial {f}_{\mathrm{DC}}}{\partial {\rho }^{H}}={F}^{H}\varphi F$$

A fourfold cross-validation was performed on the 20 healthy subjects resulting in 15 healthy subjects for training and five held-out subjects for validation and testing. Further testing included 3D cardiac CINE from 15 patients (not seen in training). We trained CINENet on retrospectively undersampling the isotropic 3D CINE reference acquisition (2.5x) of healthy subjects with randomly selected acceleration factors in the range of 3× to 8× and temporal resolutions per cardiac phase in the range of 16–78 ms, resulting in 6,500 4D volumes and providing a range of aliasing artifact impact and temporal resolutions. Every 6,500 iterations, new sampling masks were created and applied to the reference images. Training was performed with ADAM optimizer for a mean-squared error loss over 50 epochs with early stopping and learning rate of ${10}^{-4}, {\beta }_{1}=0.9, {\beta }_{2}=0.999, \epsilon = {10}^{-8}$, fixed data consistency $\lambda =0.01$ for all data consistency blocks and batch size of 44.

### In-vivo data acquisition

Imaging was performed on a 1.5 T MRI scanner (MAGNETOM Aera, Siemens Healthcare, Erlangen, Germany) equipped with 18-channel body and 32-channel spine coils. Written informed consent was obtained from all subjects and the study was approved by the local ethics committee (healthy subjects: London Bridge Research Ethics Committee, patients: North of Scotland Research Ethics Committee). All experiments were performed in accordance with the relevant guidelines and regulations.

The prospectively ECG-triggered 3D Cartesian balanced steady-state free precession (bSSFP) sequence with CINE VD-CASPR sampling and LV coverage was acquired in 20 healthy subjects (11 females, age = 33 ± 5 years) and in 15 patients (5 females, age = 48 ± 13 years). For each healthy subject, four acquisitions (one reference and three prospectively undersampled) were performed in short-axis orientation with isotropic 1.9 mm^3^ resolution covering a FOV of 304 × 213 × 84 mm^3^ (left–right × superior–inferior × anterior–posterior). For the reference scan with acceleration 2.5×, due to the required extended breath-hold, the acquired resolution was 1.9 × 1.9 × 3.8 mm which was reconstructed to 1.9 mm^3^ isotropic resolution. In patients, one prospectively undersampled acquisition with slightly anisotropic resolution of 1.9 × 1.9 × 2.5 mm covering a FOV of 304 × 213 × 110 mm^3^ (LR × HF × AP) was performed. Remaining imaging parameters were similar and included: echo time (TE) = 1.3 ms, repetition time (TR) = 2.6 ms, flip angle = 39°, bandwidth = 1,042 Hz/px, phase oversampling = 15%, slice oversampling = 20% and fully sampled k-space center $c\hspace{0.17em}$= 15%. In each acquisition ${N}_{C}=16$ cardiac phases were acquired and the number of segments per spiral arm R = 14–22 was adapted to fit within the subject’s cardiac cycle (50–91 bpm, 68 ± 16 bpm) which determined the temporal resolution *R*·TR= 38–56 ms (45 ± 4 ms). Acquisition times in healthy subjects were 30 ± 2 s for the reference acquisition with an acceleration factor of 2.5× and 12 ± 1 s/10 ± 1 s/7 ± 1 s for the prospectively undersampled acquisitions with accelerations of 9×/11×/15×, respectively. In patients, a scan time of 10 ± 1 s was achieved for the prospectively undersampled acquisition with acceleration 12×.

A multi-slice SA 2D bSSFP CINE acquisition with retrospective gating and 2× GRAPPA acceleration was performed for all healthy subjects and patients. 2D CINE was acquired in eight breath-holds of 15 s duration (two slices per breath-hold) each with 20 s pause in between, resulting in an acquisition time of 4 min 20 s. 2D CINE acquisition parameters included: in-plane resolution of 1.9 × 1.9 mm (acquired and reconstructed), slice thickness of 8 mm, temporal resolution of ~ 40 ms, 20 cardiac phases (reconstructed), TE = 1.06 ms, TR = 2.12 ms, flip angle = 52° and bandwidth = 915 Hz/px and similar FOV as for 3D CINE.

### Evaluation

The proposed CINENet is compared against a CS reconstruction with ${\mathcal{l}}_{1}$-regularized spatial Haar wavelets and temporal TV regularization^13^ along the cardiac phases. Regularization parameters were carefully optimized over all datasets (same parameters for all subjects and undersampling factors) and were ${\lambda }_{s}=0.001$ for spatial direction and ${\lambda }_{t}=0.005$ for temporal direction. A GPU-accelerated CS reconstruction using the BART toolbox^58^ was employed. Reconstruction times are reported excluding coil sensitivity map generation time.

Image quality was quantitatively assessed by myocardium-to-blood contrast ratios (CR) in end-systolic and end-diastolic cardiac phases and was measured as

9 $${\text{CR}} = \frac{{{\text{mean}}\;\left( {{\text{ROI}}_{{{\text{myocardium}}}} } \right)}}{{{\text{mean}}\;\left( {{\text{ROI}}_{{{\text{blood}}}} } \right)}}$$

with blood pool region of interests (ROIs) drawn in left and right ventricle blood pool for 2D CINE and 3D CINE reconstructed with CINENet and CS. We drew ROIs at similar anatomical positions of 2D and 3D CINE in apical, mid-apical and basal slices. We report average and standard deviation over all test subjects, measurements and systolic and diastolic phase. Statistical significance for CR was determined with a paired Welch’s t-test (significance level of $$P<0.05)$$ and Bonferroni correction under the null hypothesis of equal means for unequal variances.

Quantitative LV function assessment was conducted with end-systolic volume (ESV), end-diastolic volume (EDV) and ejection fraction (EF). LV epicardial and endocardial segmentation masks of 2D CINE and 3D CINE acquisitions were automatically determined with the Segment software^59^ and afterwards manually corrected.

## Supplementary information

Supplementary Information.

Supplementary Video S1.

Supplementary Video S2.

Supplementary Video S3.
