# Supplementary material for: CINENet: deep learning-based 3D cardiac CINE MRI reconstruction with multi-coil complex-valued 4D spatio-temporal convolutions
Source: Sci Rep. 2020 Aug 13;10:13710. doi: 10.1038/s41598-020-70551-8 (PMC7426830; doi:10.1038/s41598-020-70551-8)
Supplement: Supplementary file 1 — Supplementary Information. [file 41598_2020_70551_MOESM1_ESM.docx]

**CINENet: Deep Learning-based 3D Cardiac CINE MRI Reconstruction with Multi-coil Complex-valued 4D Spatio-Temporal Convolutions**

**Authors**

Thomas Küstner^1^, Niccolo Fuin^1^, Kerstin Hammernik^2^, Aurelien Bustin^1^, Haikun Qi^1^, Reza Hajhosseiny^1^, Pier Giorgio Masci^1^, Radhouene Neji^1,3^, Daniel Rueckert^2^, René M Botnar^1,4^, Claudia Prieto^1,4^

**Affiliations**

^1^School of Biomedical Engineering and Imaging Sciences, King’s College London, St. Thomas’ Hospital, London, United Kingdom.

^2^Department of Computing, Imperial College London, London, United Kingdom.

^3^MR Research Collaborations, Siemens Healthcare Limited, Frimley, United Kingdom.

^4^Escuela de Ingeniería, Pontificia Universidad Católica de Chile, Santiago, Chile

**Corresponding Author, Reprint Info**

Thomas Küstner, School of Biomedical Engineering and Imaging Sciences, King’s College London, St. Thomas’ Hospital, Lambeth Wing, London, United Kingdom. E-Mail: thomas.kuestner@kcl.ac.uk

**Running Title**

4D Cardiac MRI CINENet

**Word count**

3505 words

# SuppLEMENTARY MATERIAL


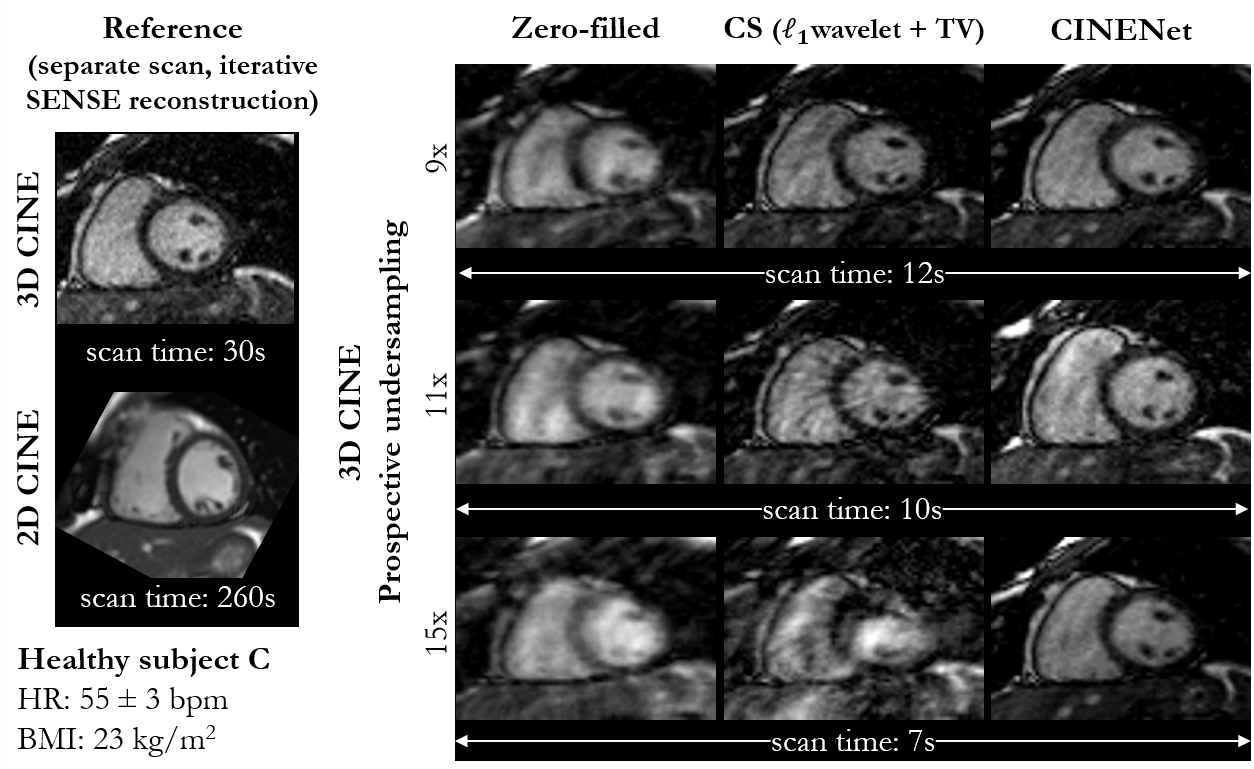


Supplementary Figure 1: End-diastolic, mid-apical images in short axis of a healthy subject with normal heart rate. Images are acquired with prospectively undersampled single breath-hold 3D Cartesian CINE with isotropic (1.9mm^3^) left ventricle coverage for an acceleration of 9x (scan time 12s), 11x (scan time 10s) and 15x (scan time 7s) in comparison to ground-truth reference (separate acquisition) of single breath-hold 3D CINE (2.5x, scan time 30s) and conventional multi breath-hold 2D CINE (2x, scan time 260s). The reference 3D CINE is reconstructed with iterative SENSE. Undersampled 3D CINE images are reconstructed with coil-weighted zero-filling (network input), Compressed Sensing (CS) with L1-regularized spatial wavelets and temporal total variation (TV) and with the proposed CINENet.


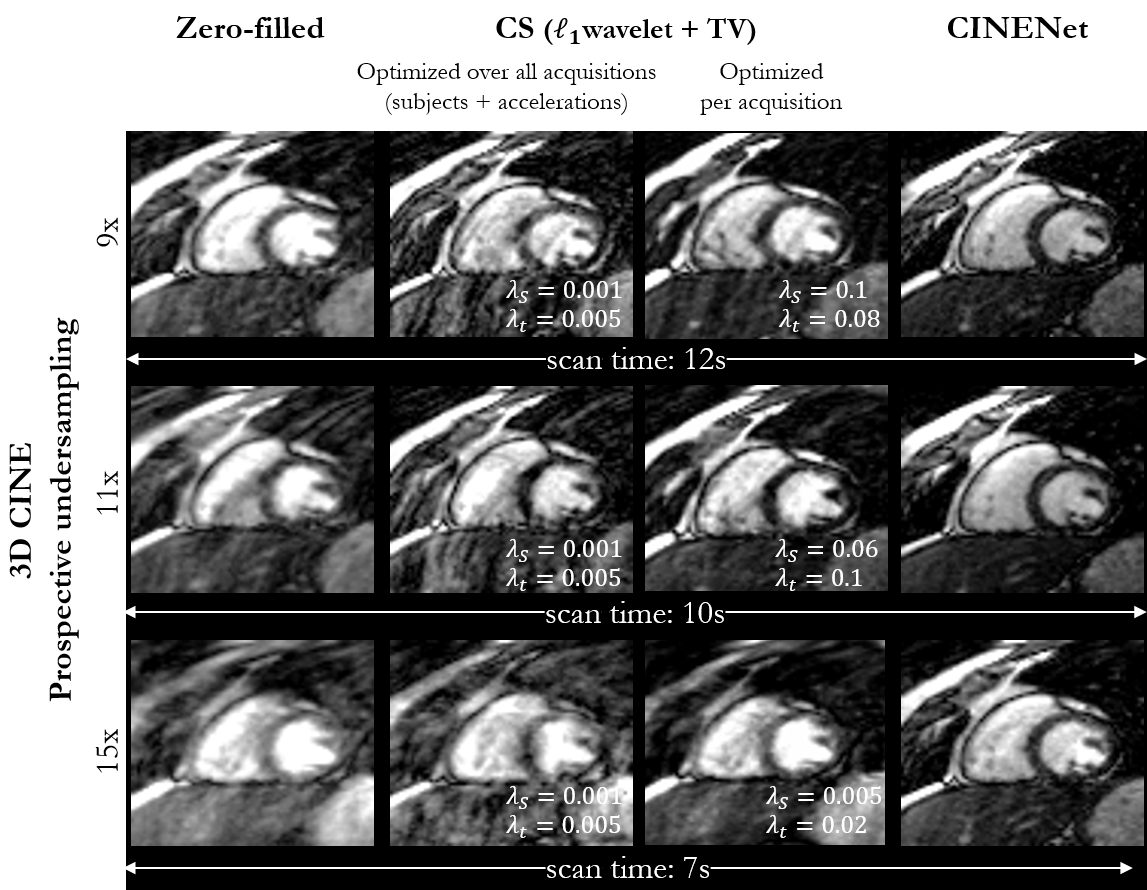


Supplementary Figure 2: End-diastolic, mid-apical images in short axis of healthy subject A. Images are acquired with prospectively undersampled single breath-hold 3D Cartesian CINE with isotropic (1.9mm^3^) left ventricle coverage for an acceleration of 9x (scan time 12s), 11x (scan time 10s) and 15x (scan time 7s). Undersampled 3D CINE images are reconstructed with coil-weighted zero-filling (network input), Compressed Sensing (CS) with L1-regularized spatial wavelets and temporal total variation (TV) and with the proposed CINENet. The CS regularization parameters were either optimized over all acquisitions (complete cohort and all acceleration factors) or individually for each acquisition.


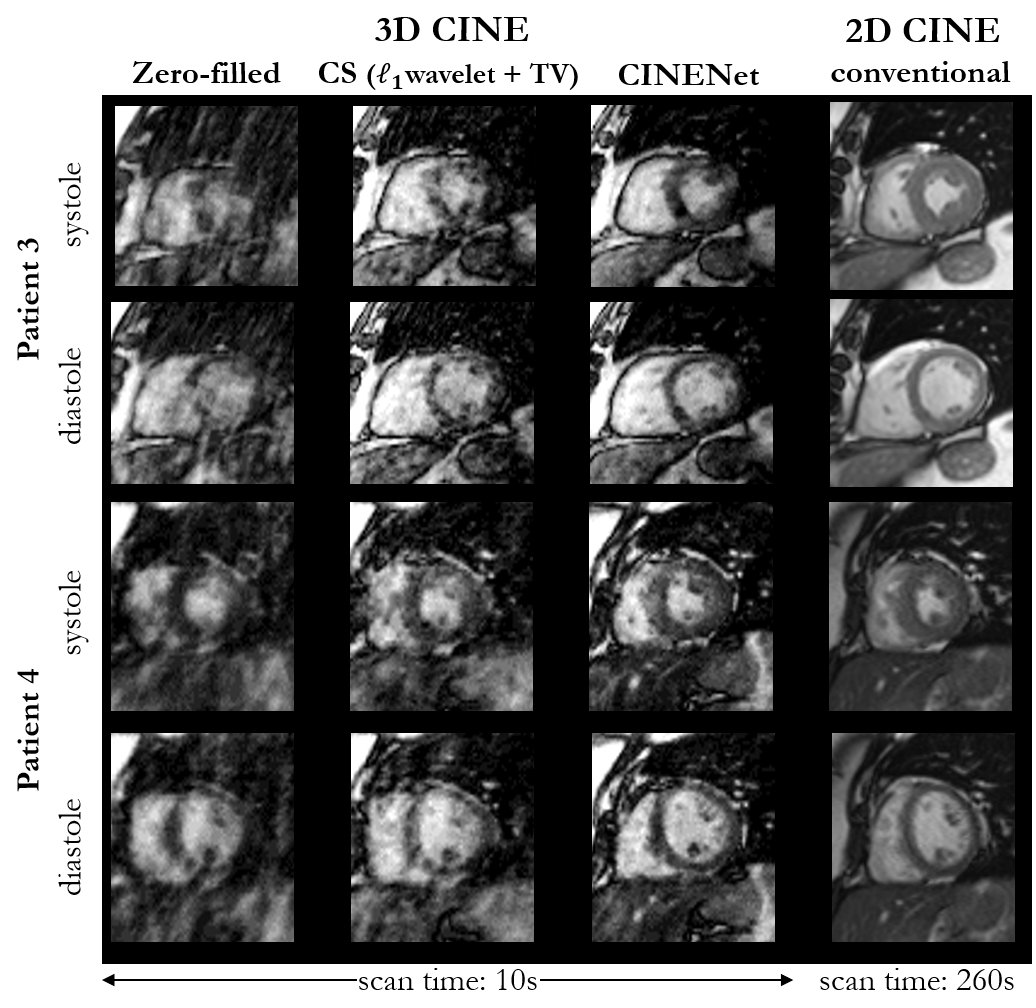


Supplementary Figure 3: End-systolic and end-diastolic mid-apical images in short axis of two patients with suspected cardiovascular disease (Patient 3: dilated cardiomyopathy, Patient 4: myocarditis). Images are acquired with prospectively undersampled single breath-hold 3D Cartesian CINE with slightly anisotropic (1.9x1.9x2.5mm) left ventricle coverage for an acceleration of 12x (scan time 10s) in comparison to conventional multi breath-hold 2D CINE (2x, scan time 260s). Undersampled 3D CINE images are reconstructed with coil-weighted zero-filling (network input), Compressed Sensing (CS) with L1-regularized spatial wavelets and temporal total variation (TV) and with the proposed CINENet.


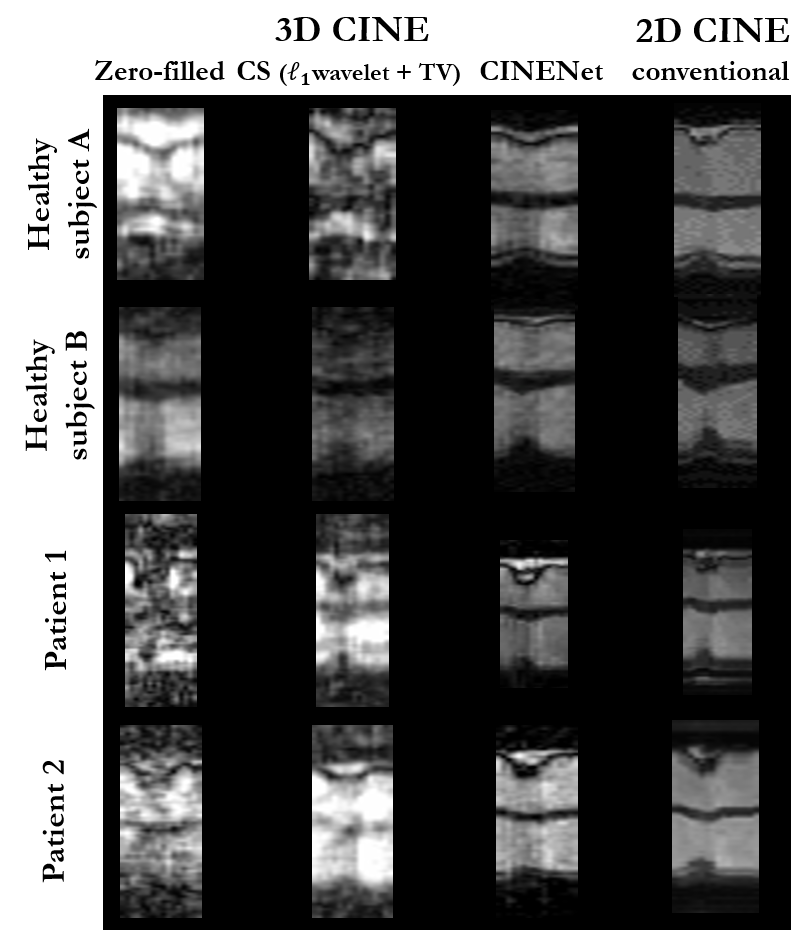


Supplementary Figure 4: Temporal profiles in mid-ventrical position for two healthy subjects and two patients of the prospectively undersampled 3D CINE (15x accelerated for healthy subjects and 12x accelerated for patients) in comparison to 2D CINE.


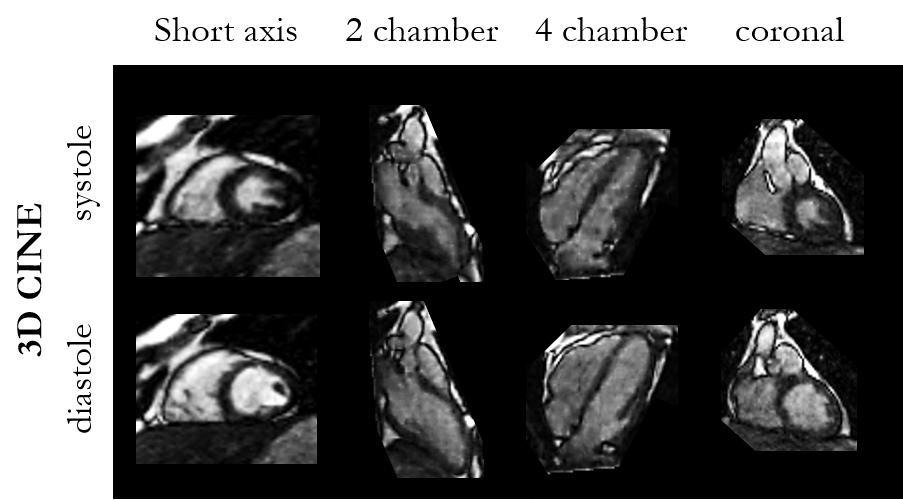


Supplementary Figure 5: End-systolic and end-diastolic reformatted images in vertical long axis (2 chamber), horizontal long axis (4 chamber) and coronal orientation of the short axis acquisition in a healthy subject. Images are reconstructed with the proposed 4D CINENet from the 15x accelerated single breath-hold 3D Cartesian CINE (scan time 7s) acquisition.


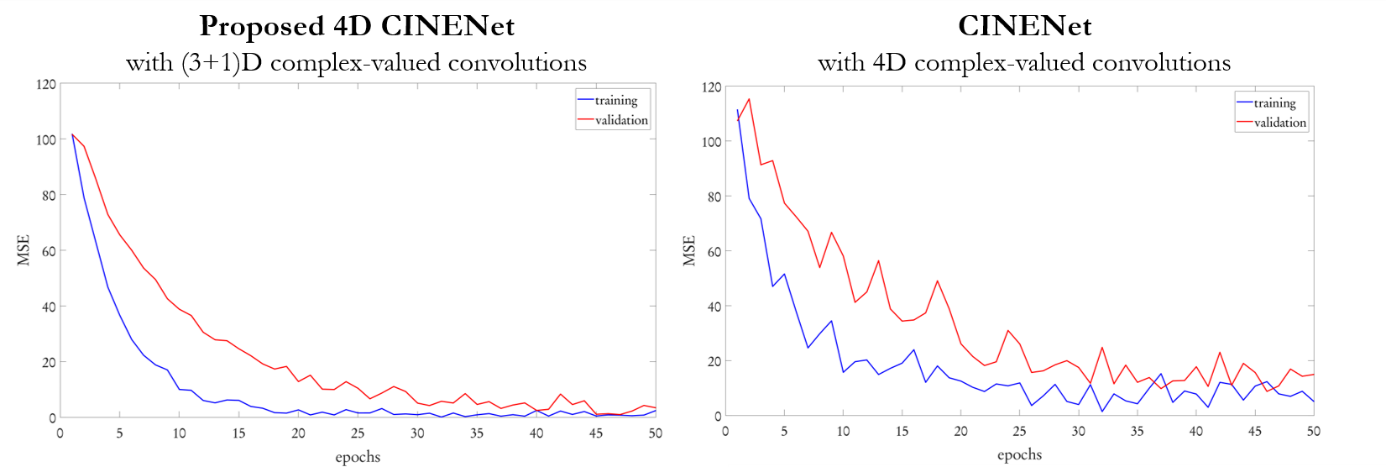


Supplementary Figure 6: Training and validation mean-squared error (MSE) loss over training epochs. Comparison between proposed (3+1)D complex-valued convolutions and full 4D complex-valued convolutions.

Supplementary Video S1: Cardiac motion-resolved mid-apical images in short axis of a healthy subject acquired with prospectively undersampled single breath-hold 3D Cartesian CINE with isotropic (1.9mm^3^) left ventricle coverage for an acceleration of 9x (scan time 12s), 11x (scan time 10s) and 15x (scan time 7s) in comparison to ground-truth reference (separate acquisition) of single breath-hold 3D CINE (2.5x, scan time 30s) and conventional multi breath-hold 2D CINE (2x, scan time 260s). Reference images are reconstructed with iterative SENSE. Undersampled 3D CINE images are reconstructed with coil-weighted zero-filling (network input), Compressed Sensing (CS) with L1-regularized spatial wavelets and temporal total variation (TV) and with the proposed CINENet.

Supplementary Video S2: Cardiac motion-resolved images in short axis ranging from base to apex of one healthy subject acquired with 15x accelerated single breath-hold 3D Cartesian CINE (scan time 7s) and reconstructed with the proposed 4D CINENet in comparison to conventional multi-slice and multi breath-hold 2D CINE (scan time 260s). For 3D CINE, similar anatomical slice locations to 2D CINE have been selected, i.e. not all 3D CINE slices are shown. Spatial coverage is depicted in Supplementary Video S3.

Supplementary Video S3: Spatial left ventricular coverage in short axis ranging from base to apex of one healthy subject acquired with 15x accelerated single breath-hold 3D Cartesian CINE (scan time 7s) and reconstructed with the proposed 4D CINENet in comparison to conventional multi-slice and multi breath-hold 2D CINE (scan time 260s).
